# An update on pediatric endoscopy

**DOI:** 10.1186/2047-783X-18-24

**Published:** 2013-07-25

**Authors:** Michael Friedt, Simon Welsch

**Affiliations:** 1 Department of General Pediatrics, Neonatology and Pediatric Cardiology, Division of Pediatric Gastroenterology University Children’s Hospital, Moorenstr. 5, D-40225, Duesseldorf, Germany

**Keywords:** Endoscopy, Pediatric, Colonoscopy, Child, Capsule endoscopy, Pediatric gastroenterology

## Abstract

Advances in endoscopy and anesthesia have enabled gastrointestinal endoscopy for children since 1960. Over the past decades, the number of endoscopies has increased rapidly. As specialized teams of pediatric gastroenterologists, pediatric intensive care physicians and pediatric endoscopy nurses are available in many medical centers, safe and effective procedures have been established. Therefore, diagnostic endoscopies in children are routine clinical procedures. The most frequently performed endoscopies are esophagogastroduodenoscopy (EGD), colonoscopy and endoscopic retrograde cholangiopancreaticography (ERCP). Therapeutic interventions include variceal bleeding ligation, foreign body retrieval and percutaneous endoscopic gastrostomy. New advances in pediatric endoscopy have led to more sensitive diagnostics of common pediatric gastrointestinal disorders, such as Crohn’s disease, ulcerative colitis and celiac disease; likewise, new diseases, such as eosinophilic esophagitis, have been brought to light.

Upcoming modalities, such as capsule endoscopy, double balloon enteroscopy and narrow band imaging, are being established and may contribute to diagnostics in pediatric gastroenterology in the future.

## Introduction

Since its introduction (or first descpription) in the 1960s, the field of pediatric gastroenterology has developed rapidly. Therefore, pediatric gastroenterology has become a subspecialty in many countries. The Federation of International Societies of Pediatric Gastroenterology, Hepatology, and Nutrition (FISPGHAN) is analyzing and developing the implementation of Pediatric Endoscopy worldwide and aims to introduce a standardized curriculum for trainees and training the trainers [1]. Thomson *et al*. showed the advantages of an intensive training by virtual endoscopy training [2].

During the last 30 years, the number of pediatric gastroenterologists increased from a few select centers around the world to an ever-growing specialty. In the US there is approximately one pediatric gastroenterologist per 100.000 children. With the development of a subspecialty focused on disorders of the pediatric gastrointestinal (GI) tract, new technologies such as pediatric endoscopy were developed to aid in diagnoses. Pediatric esophagogastroduodenoscopy (EGD) began in the 1970s and has evolved from an infrequent procedure in the operating room with a single ocular for viewing the GI-tract to a routine outpatient procedure using intravenous sedation and large viewing screens. Due to technical advances in gastrointestinal endoscopy and anesthesia, even premature infants and severely sick patients can be examined from the first day of birth on.

The most frequently performed, mainly diagnostic, procedures are EGD and colonoscopy. Wireless capsule endoscopy (CE) or double balloon enteroscopy for investigation of the small intestine can be performed alternatively to magnetic resonance (MR) enteroclysis. The former has been done in infants during the first year of life. On the other hand, therapeutic procedures, such as polypectomy, retrieval of foreign bodies, percutaneous endoscopic gastrostomy (PEG) placement, endoscopic retrograde cholangiopancreaticography (ERCP) or ligation of esophageal varices can now be performed during early infancy in the neonatal period. In contrast to adults, endoscopic examinations in children are usually performed under deep sedation or general anesthesia to reduce emotional stress caused by separation from parents and the preparation for the procedure itself.

In children, gastrointestinal endoscopy is usually performed by specialized pediatric gastroenterologists. Starting in 1999 guidelines for trainees to ensure competence in the field of pediatric gastroenterology and training pediatric endoscopy have been issued by the North American Society of Pediatric Gastroenterology and Nutrition (NASPGHAN), the European Society of Paediatric Gastroenterology Hepatology and Nutrition (ESPGHAN) and by many national boards [1,3].

## Review

### Patient and parent preparation

Preparation for gastrointestinal endoscopy in children should respect the special physiology as well as the psychosocial and emotional needs of pediatric patients and their parents. It is recommended that the preparation should start early. Prior to elective procedures, informed consent of the parents or guardians has to be obtained. Parents and children should be provided with sufficient information about potential risks and benefits of the procedure using an age-appropriate language. The important elements of this discussion should be acknowledged in writing and signed to provide legal documentation.

### Preprocedure assessments

Preprocedure assessment includes a systematic review and physical examination with a special focus on the airway system. Examination and documentation of loose teeth, oral piercings and enlarged tonsils is very important. Loose teeth can be accidentally dislodged and may cause complications when entering the airways. Enlarged tonsils can provoke breathing difficulties and obstructive apnea in sedated patients and should be evaluated by the physician before sedation. Laboratory tests may include coagulation and liver function tests; however, the significance of pre-endoscopic routine coagulation screening is limited [4]. Endoscopic procedures are contraindicated in most cases of severe coagulopathy and adequate treatment has to be provided, if endoscopy is necessary.

To reduce anxiety in the younger patients, the presence of parents for preprocedural preparation is usually essential [5]. Premedication with benzodiazepines has been shown to reduce anxiety and fear prior to endoscopy. Oral or nasal application is possible [6]. Liacouras *et al*. showed that midazolam can also reduce the emotional stress of separation from the parents and makes the patients feel more comfortable during intervention. Additionally, there was no significant difference with regard to the duration of the procedure, surveillance parameters, length of hospital stay and the recovery time in the midazolam group [7].

### Dietary restrictions

Preprocedural fasting and intestinal preparation depends on patient age and the planned procedure. Traditionally, it is recommended that patients fast from solids for six hours and from liquids for two to four hours. Splinter *et al*. examined the difference of stomach volume and pH during conventional prolonged fasting with the allowance of clear fluid intake up to two to three hours before sedation. They showed that there was no significant difference between the different treatment groups [8]. A longer fasting time may be required for conditions such as gastric outlet obstruction and achalasia because retained food may increase the risk of aspiration. The guideline of the American Academy of Pediatrics (AAP) on sedation follows the recommendations of the American Society of Anesthesiologists (ASA): children should be offered clear liquids (including breast milk but not formula milk) up to two to three hours before sedation to avoid dehydration. Infants younger than six months may receive infant formula up to four to six hours and clear liquids up to two hours before sedation. Patients older than six months should be fasting from nonclear liquids and solids for six to eight hours before sedation [9,10].

### Antibiotic prophylaxis

According to the guidelines of the American Heart Association (AHA) and the American Society of Gastroenterological Endoscopy (ASGE) antibiotic prophylaxis is recommended only for specific conditions. These include cardiac lesions with high or moderate risk of bacterial endocarditis. In addition to heart diseases, the following conditions favor an antibiotic prophylaxis: neutropenia, ventriculoperitoneal shunts or therapeutic interventions (for example, PEG-tube placement, sclerotherapy, stricture dilatations) [11]. However, antibiotic recommendations are not standardized, with the ASGE proposing prophylaxis only in high-risk patients.

### Contraindications

Absolute contraindications include unstable airway, cardiovascular collapse, intestinal perforation and peritonitis. Relative contraindications are bowel obstruction, severe thrombocytopenia, coagulopathy, recent gastrointestinal surgery, respiratory infections and recent food intake. The procedure should be delayed or cancelled in the absence of signed consent.

### Sedation

EGD and colonoscopy in children are generally performed under moderate sedation (conscious sedation) or general anesthesia. Advantages of moderate sedation are remaining protective airway reflexes and spontaneous breathing during examination. On the other hand, deep sedation provides a more reliable state of sedation. Pediatric patients are more likely to have respiratory complications because of their higher lung resistance. Infants less than seven months are at higher risk due to obligatory nasal breathing. Additionally, children are less resistant against hypoxemia. Respiratory infections in children with known hyperactive airways are an absolute contraindication for elective endoscopy in sedation. In most cases deep sedation or general anesthesia is necessary. According to the most recent analysis and guidelines, propofol-based sedation seems to be the safest and most convenient method of inducing a sufficient sedation [12].

### Patient monitoring

In most hospitals an interdisciplinary team of pediatric gastroenterologists, pediatric intensivists or anesthetists and specialized nurses is involved in the endoscopy procedures. All patients should be monitored regarding the cardiovascular system including oxygen-saturation. Monitoring should be continued for 15 to 30 minutes after the procedure. Afterwards the patient should stay on the ward for at least two hours. Intake of clear fluid is possible one hour after sedation. Discharge is possible if sufficient cardiovascular function and airway patency is confirmed, the patient is fully oriented and protective reflexes are intact [13].

### Equipment

Basic equipment includes emergency equipment for children of all ages, such as intravenous lines, laryngoscopes, tubes, masks and nasogastric tubes. Endoscopes must be chosen regarding the age of the pediatric patient. Additionally, catheters have to be provided to solve possible complications and make unexpected treatments. The gastroscopes used in adults can be used in children above 25 kg [14]. Smaller endoscopes (5 to 8 mm) are appropriate for smaller children. Colonoscopes used in adults (11 to 13 mm) may be used in adolescents, whereas in very small patients colonoscopies can be performed with slim gastroscopes. In these cases colonoscopy has to be performed with extreme precaution as the higher stiffness of gastroscopes may lead to a higher risk of perforation. Additionally, catheters of different calibers depending on the thickness of the endoscope have to be provided.

### Esophagogastroduodenoscopy

In parallel with the increase in pediatric EGD procedures since the 1970s, the incidence of disorders that require EGD for diagnosis in children has increased. Franciosi *et al*. have shown that subject characteristics and endoscopy practices during a 20-year interval have changed [15]. There was a 12-fold increase in the number of first-time EGDs performed from 1985 to 2005. This may lead to an increasing incidence of disease rates. However, an increase of disease rates may instead reflect increasing rates of disease diagnosis rather than a true rise of disease occurrence. The inclusion of children with less severe clinical presentations and the collection of greater numbers of biopsies per procedure might play an influencing role. During the 20-year interval the proportion of patients with gastrointestinal bleeding was reduced from 34% to 5%, whereas the proportion of subjects with abdominal pain increased from 23% to 43%. Additionally, the rate of complete EGD (biopsies from the esophagus, stomach and duodenum) increased from 18% in 1985 to 95% in 2005. Technical improvements and physician’s technical experiences have led to new discoveries and interests in pediatric GI inflammatory disorders which might have also influenced the number of EGDs performed. In particular, eosinophilic esophagitis (EoE) has received considerable attention as a ‘new’ disease, with the first consensus report being published in 2007. EoE is a disorder, which requires EGD with biopsy for diagnosis. The sensitivity of detecting EoE is dependent on the number of esophageal biopsies taken at the time of diagnosis. The sensitivity is only 55%, if only one biopsy is taken, compared to 94% if ≥4 biopsies are taken [16], indicating that fewer biopsies underestimate the true incidence and prevalence of this disorder. Furthermore, the recognition of EoE as a distinct clinical entity from gastroesophageal reflux disease (GERD) and functional abdominal pain has increased the use of pediatric EGD in the last decade as an important diagnostic modality for a disorder that can otherwise not be diagnosed. Celiac disease is another important pediatric GI disorder in which endoscopy is the gold standard used to establish the diagnosis. A dramatic rise in the incidence rate of celiac disease has been reported from 0.9/100,000 in 1950 to 9.1/100,000 in 2001. The estimated prevalence of celiac disease in subjects without risk factors is as high as 1:133 (0.8%) in the US [17]. Celiac disease has often been described as an iceberg: subjects with severe symptomatic disease are more likely to go to their physician and have their diagnosis of celiac disease confirmed by duodenal biopsy represent only a small fraction of the true population with milder or even asymptomatic disease.

According to recent guidelines EGD is recommended for all patients suspected of inflammatory bowel disease (IBD) irrespective of the presence or absence of upper gastrointestinal symptoms [18,19]. In contrast to previous publications, Kugathasan *et al*. reported pediatric population-based IBD incidence rates with a higher proportion of children with Crohn disease [20]. IBD detection rates may, therefore, be confounded by the changing practices of pediatric EGD. EGD for possible oncologic diseases of the upper GI-tract in children is rare. However, investigation of graft-versus-host-disease (GvHD) is a common indication for EGD after bone marrow transplantation [21].

Although most EGDs are performed due to diagnostic indications, there are a few therapeutic procedures, which have also increased during the last decades:

Foreign bodies. Removal of ingested foreign bodies is urgent if the swallowed objects are found in the esophagus. Additionally, food bolus impaction due to strictures, stenosis or EoE is an indication for urgent removal. Ingested foreign bodies, which have passed the esophagus, will pass the GI-tract in most cases. However, sharp or toxic foreign bodies, for example batteries, have to be removed. Regardless of the properties of the ingested object EGD is indicated if passing is delayed and foreign bodies remain in the stomach. Additionally, harmless appearing objects might induce tissue-damage. Ingestion of multiple magnets (for example, from toys, jewelry) may cause intestinal obstruction and perforation, so that immediate retrieval is indicated after ingestion of these objects. Creating awareness is necessary since the number of emergency retrievals of magnets has risen in the last few years. Most ingested foreign bodies are coins, batteries and toys. Button batteries are the most dangerous parts, because the risk of tissue necrosis in the esophagus is particularly high. Batteries can lead to severe esophageal damage and fistula formation even days after their removal indicating the urgency of foreign body retrieval [22].

Gastrostomy. Due to its good outcome, PEG placement has become widely accepted in infants and children needing long-term tube-feeding. However, it has well-recognized complications [23]. According to recent recommendations, enteral nutrition exceeding four to six weeks is an indication for gastrostomy or enterostomy [24].

Endoscopic treatment of gastroesophageal reflux disease in children with endoluminal gastroplication can be performed in specialized centers with a good outcome [25]. Treatment of upper gastrointestinal bleeding caused by portal hypertension includes variceal band ligation and sclerotherapy, which are both safe and effective techniques in children and can be done even in small infants [26].

### Colonoscopy

The safety and effectiveness of colonoscopy in the detection of lower GI-tract pathology in children has been established during the last three decades. Skills and experience have advanced to the point that both diagnostic and therapeutic colonoscopies are now routinely performed by most pediatric gastroenterologists. The available equipment permits examination of all pediatric patients including neonates. Successful completion including ileal intubation is a technical challenge among all pediatric patients. An additional level of complexity in pediatric patients is the poor compliance with the necessary bowel cleansing and the difficulties in sedating a frightened or otherwise uncooperative patient.

Pre-procedural preparation should be individualized according to the child’s age, cooperation of the child and the individual experience of the specific center. In infants, adequate preparation can usually be obtained with the use of small-volume enemas and by substituting clear liquids for milk 12 to 24 hours prior to the procedure. Since there is no ideal bowel cleansing regimen in children, various protocols have been compared by Turner *et al*. Several evidence-based protocols were proposed to optimize preparation and minimize adverse effects [27]. The acute toxicity rate of oral sodium phosphate was estimated to be at most 3/7,320 colonoscopies (0.041%). The safety and effectiveness of large polyethylene glycol-based solutions with electrolytes (PEG-ES), causing osmotic diarrhea, has been demonstrated. Nevertheless, taste and volume might be barriers to efficient colonoscopy preparation. In the combination of polyethylene glycol 3350 with a sports drink nausea/ vomiting were the most reported side effects followed by abdominal pain/cramping and fatigue/weakness [28,29]. Continuous application via a nasogastric tube might improve tolerability in some of the children. Recently, the safety and efficacy of a two-day small volume electrolyte-free preparation (PEG-P) has been reported, which, additionally, was well tolerated and might improve compliance in the near future [30].

The indications for diagnostic colonoscopy in children are basically similar to the ones in adults. As shown, the main causes leading to colonoscopy in children are hematochezia, abdominal pain and diarrhea. The most common endoscopic diagnoses are IBD (diagnosis or review), juvenile polyps (with polypectomy), polyposis syndromes (diagnosis or review), allergic colitis and miscellaneous (vascular anomaly, infective colitis, tumors or GvHD) [31,32]. However, since IBD presents in 25% to 30% of patients before the age of 20 years and polyps are the most common causes of rectal bleeding in children, these two diagnostic categories are the most common diagnoses in pediatric lower gastrointestinal disorders.

Absolute contraindications to colonoscopy in pediatric patients are suspected bowel perforation and acute peritonitis. The safety and effectiveness of pediatric colonoscopy has been demonstrated in a few reports. Bleeding after colonoscopy is usually minimal but may occur after mucosal biopsy or polypectomy. Depending on the case series, bleeding occurs in 0.26% to 2.5% of patients after colonoscopy. Colonic perforation is the most serious complication of colonoscopy in children and is usually related to polypectomy. Its risk ranges from 0.06% to 0.3%. Bacteremia is rare even after polypectomy and multiple biopsies.

### Endoscopic retrograde cholangiopancreatography

Since the first report of successful cannulation of the ampulla of Vater in 1968, ERCP has been widely used in the management of pancreatic and heptobiliary disorders in adults. The first successful ERCP in a 3.5-month-old child using an adult size duodenoscope was reported by Waye in 1976 [33]. Since the development of smaller diameter duodenoscopes in the 1980s and 1990s, the field of pediatric endoscopy has grown considerably and several retrospective reports have demonstrated the safety of diagnostic and therapeutic interventions in children [34]. Its feasibility and benefit in the diagnostic workup of neonatal cholestasis has been shown in recent years [35]. Due to the results of the ERCP, a surgical exploration and cholangiogram was not necessary in 25% of infants with suspected biliary atresia [36]. However, its superiority as compared with the other types of cholangiograms remains to be demonstrated in the assessment of patients with neonatal cholestasis.

Recent advantages in magnetic resonance imaging have led to the development of a novel technique: magnetic resonance cholangiopancreatography (MRCP). MRCP is used more and more often as a diagnostic tool in patients with pancreaticobiliary disorders. The possibility of endoscopic intervention (e.g. papillotomy, retrieval of biliary stones) is is a major advantage of ERCP in comparison to MRCP. Additionally ERCP has been proven valuable and safe in children of all ages.

### Capsule endoscopy

Since its introduction and approval by the US Food and Drug Administration for children ≥10 years of age in 2003, wireless CE has been increasingly used in eligible patients [37]. Supported by experience in children as young as 10 months of age, the FDA expanded the role for CE use to children two years and older and also approved the use of a patency capsule [38].

The established indication for CE is the evaluation of small intestinal pathology for a variety of clinical conditions: unexplained gastrointestinal bleeding, small bowel Crohn’s disease (CD), small bowel tumors (polyps, neoplasms) and a broad range of miscellaneous abnormalities (for example, Henoch-Schönlein Purpura, lymphangiectasia). CE has the potential to be particularly valuable in pediatrics, as it avoids ionizing radiation, deep sedation or general anesthesia. However, the experience of the past years showed that the ability to swallow the capsule is most often the limiting factor for the feasibility of CE. The advantage of avoiding deep sedation is lost when the capsule has to be placed by EGD.

A recent review and meta-analysis showed positive small bowel findings in 58% to 72% of patients after CE, which is comparable to findings in adults. The retention rate was between 2.2% and 2.4%, which is slightly above the rate of 1.4% in adult patients (n = 22,840) [38]. The risk for potential CE retention includes known IBD, especially with small bowel involvement. However, the availability of a patency capsule (PC) may even lower the potential risk of capsule retention by using the PC in high-risk patients (known or suspected CD with possibly obstructive symptoms, such as nausea). Published studies show that CE can be safe and effective in very small pediatric patients (as small as 11.5 kg and 1.5 years of age) and the capsule can be swallowed by a majority of patients, even preschool children. The rate of incomplete studies using CE has been shown to be more frequent in pediatric patients. However, the diagnostic achievement/priority is high under these circumstances and might even improve with advanced technology and capsules with longer battery life.

### Double balloon enteroscopy

Double balloon enteroscopy (DBE) is a newly developed endoscopic modality for diagnostic and therapeutic procedures of small bowel disorders [38]. There are only limited data about the application of DBE in children and adolescents. Although DBE appears to be a safe endoscopic modality for children, it is quite invasive and should be selectively reserved for patients with a high suspicion for small intestinal pathology, in which other invasive techniques have failed to adequately diagnose and treat a patient’s disease.

### Narrow band imaging and chromoendoscopy

Narrow band imaging provides high resolution imaging of the mucosa using optical filters and light wavelengths of narrow bands to enhance the microvasculature of mucosal surfaces. This technique can be used simultaneously with endoscopy to detect early changes in the microvasculature and mucosal abnormalities in dysplastic lesions (for example, high grade dysplasia in ulcerative colitis) or early detection of Barrett’s esophagus. This newer technique might replace the modality of chromoendoscopy, which features visualization of abnormal mucosa during conventional endoscopy using dyes. The relevance of both techniques in pediatric routine clinical diagnosis has to be shown in future studies.

## Conclusions

Pediatric gastrointestinal endoscopy is a field that has been evoling in the last decades and provides a safe and effective diagnostic tool. Currently children of all ages including premature newborns can be examined, enabling more sensitive diagnoses of well known diseases (for example, IBD, celiac disease), emerging disorders (for example, EoE) and challenging diseases (for example, ERCP in neonatal cholestasis). Upcoming modalities, such as capsule endoscopy, have been proven to be safe and effective and are used more and more in pediatric patients. International and national boards for pediatric gastroenterology have been founded and have issued numerous guidelines and trainee programs for specialized pediatric gastroenterologists/endoscopists improving medical care for children with disorders of the gastrointestinal tract.

## Abbreviations

AAP: American Academy of Pediatrics; AHA: American Heart Association; ASA: American Society of Anesthesiologists; ASGE: American Society of Gastroenterological Endoscopy; CD: Crohn’s disease; CE: Capsule endoscopy; DBE: Double balloon enteroscopy; EGD: Esophagogastroduodenoscopy; EoE: Eosinophilic esophagitis; ERCP: Endoscopic retrograde cholangiopancreaticography; ESPGHAN: European Society of Paediatric Gastroenterology Hepatology and Nutrition; FAP: Familial adenomatous polyposis; FISPGHAN: Federation of International Societies of Pediatric Gastroenterology Hepatology and Nutrition; GERD: Gastroesophageal reflux disease; GI: Gastrointestinal; GvHD: Graft-versus-host-disease; IBD: Inflammatory bowel disease; MRCP: Magnetic resonance cholangiopancreatography; MRI: Magnetic resonance imaging; NASPGHAN: North American Society of Pediatric Gastroenterology Hepatology and Nutrition; PC: Patency capsule; PEG: Percutaneous endoscopic gastrostomy; PEG-ELS: Polyethylene-glycol with electrolyte solution.

## Competing interests

The authors declare that they have no competing interests.

## Authors’ contributions

MF and SW equally conceptualized this article, sampled publications and completed the manuscript. Both authors read and approved the final manuscript.
